# Rheological Characterization of the Bundling Transition in F-Actin Solutions Induced by Methylcellulose

**DOI:** 10.1371/journal.pone.0002736

**Published:** 2008-07-16

**Authors:** Simone Köhler, Oliver Lieleg, Andreas R. Bausch

**Affiliations:** Lehrstuhl für Biophysik E27, Technische Universität München, Garching, Germany; The University of Manchester, United Kingdom

## Abstract

In many *in vitro* experiments Brownian motion hampers quantitative data analysis. Therefore, additives are widely used to increase the solvent viscosity. For this purpose, methylcellulose (MC) has been proven highly effective as already small concentrations can significantly slow down diffusive processes. Beside this advantage, it has already been reported that high MC concentrations can alter the microstructure of polymer solutions such as filamentous actin. However, it remains to be shown to what extent the mechanical properties of a composite actin/MC gel depend on the MC concentration. In particular, significant alterations might occur even if the microstructure seems unaffected. Indeed, we find that the viscoelastic response of entangled F-actin solutions depends sensitively on the amount of MC added. At concentrations higher than 0.2% (w/v) MC, actin filaments are reorganized into bundles which drastically changes the viscoelastic response. At small MC concentrations the impact of MC is more subtle: the two constituents, actin and MC, contribute in an additive way to the mechanical response of the composite material. As a consequence, the effect of methylcellulose on actin solutions has to be considered very carefully when MC is used in biochemical experiments.

## Introduction

The structural organization of the cytoskeleton determines the morphology and the mechanical response of eukaryotic cells. The cytoskeleton consists of semiflexible polymers such as actin filaments which are organized into complex scaffolds by various associated actin binding proteins. Cross-linking and bundling of actin filaments are major strategies used for structural fortification of the cytoskeleton; however, filament bundling is also a governing strategy in cell motility as bundled filopodia are built up at the edge of a crawling cell [1] or actin comet tails consisting of bundled filaments propel bacteria inside their host cells [2]. Due to the biological complexity, it is a challenging task to track down the molecular origin of these dynamic processes. Therefore, *in vitro* experiments have been proven essential as reconstituted model systems enable the investigation of biochemical and physical contributions separately [3].

However, in many studies on dynamic processes Brownian motion hampers the data analysis. Increasing the viscosity of the investigated solution is helpful for reducing this problem. For this purpose, methylcellulose (MC), a polymer that forms highly viscous solutions, is widely used at concentrations of 0.2–0.5% [4]–[10]. MC is not known to specifically interact with actin or other cellular proteins; however, it has been reported that actin/MC bundles appear at MC concentrations higher than 0.3% (w/v) [11], [12], and thus biochemical experiments using MC in this concentration range have to be analyzed with care. Similarly, actin filaments can be bundled by high concentrations of polyethylene glycol (PEG) even in the absence of specific actin bundling proteins. The effect of PEG on the structure and mechanics of F-actin is purely entropic and has been studied extensively [13], [14]. At PEG concentrations below the bundling transition, depletion forces lead to increasingly effective physical cross-links giving rise to an increase in the elasticity of the actin/PEG solution [15]. Because of the higher molecular weight of MC used in this study it is a priori not clear if MC can also act as an effective depletion agent. Moreover, a mixture of two polymer solutions might give rise to additional effects influencing the structure and mechanical response of the composite material.

In contrast to the well characterized actin/PEG system, only structural information is available for actin/MC solutions [11], [12]. Here, we use macrorheology to investigate the frequency dependent mechanical response of F-actin solutions in the presence of distinct MC concentrations. Macrorheology allows quantifying the viscous and elastic part of the mechanical response of a polymer solution and therefore is a suitable tool to separate changes in solvent viscosity from alterations in the viscoelastic response of the solved polymers.

We systematically analyze the effect of increasing MC concentrations on the frequency dependent mechanical response of entangled F-actin solutions. Significant changes in the macrorheological behavior of the solution occur even at MC concentrations as low as 0.01% (w/v) – however, the structure of the actin solution remains unaffected. At concentrations higher than 0.2% (w/v) we report drastic changes in the viscoelastic response of the solution which can be traced back to filament bundling, in agreement with previous work [12].

## Results

Even in the absence of specific actin bundling proteins, actin filaments can be organized into bundles by methylcellulose as reported by [11]. Popp *et al.* have demonstrated that the bundling transition of 10 µM filamentous actin occurs at 0.3% (w/v) MC [12].

**Figure 1:**
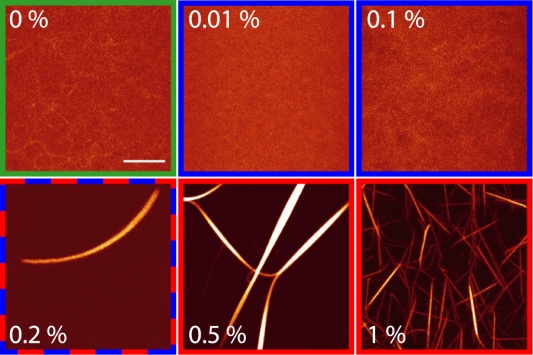
Methylcellulose induced actin bundle formation. Confocal micrographs of F-actin solutions (9.5 µM) with increasing methylcellulose concentrations. Bundles appear at a MC concentration of 0.2% (w/v), below this concentration only single filaments can be observed. At 1% MC, much more bundles occur which seem to be thinner than those formed at lower MC concentrations (scale bar: 10 µm). The color code corresponds to the three regimes depicted in figure 2.

**Figure 2:**
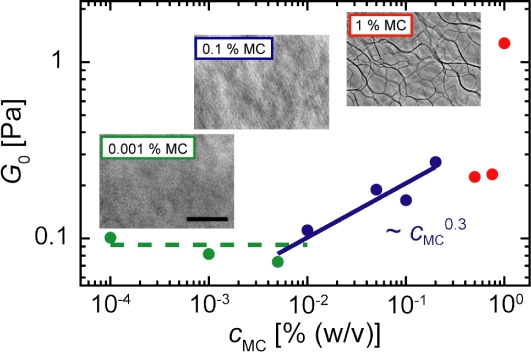
Plateau modulus of actin/MC solutions. The plateau modulus *G*_0_ of actin/MC solutions is depicted as a function of MC concentration, *c*_MC_. Three regimes can be distinguished: At low *c*_MC_ (below 0.01%, green) the plateau modulus is similar to that of an entangled F-actin solution. For intermediate *c*_MC_ (0.01–0.2%, blue) a weak increase in *G*_0_∼*c*_MC_^0.3^ is observed. Above *c*_MC_ = 0.2% (red) the dependence of *G*_0_ on *c*_MC_ becomes more complex. Insets: Phase contrast micrographs (scale bar: 20 µm).

Confocal micrographs of actin/MC gels at distinct amounts of MC are depicted in figure 1. Actin bundles cannot be observed at low (<0.01% (w/v)) and intermediate (<0.2% (w/v)) MC concentrations. However, at a critical concentration of 0.2% (w/v) actin/MC bundles start to form. Surprisingly, the thickness of the actin/MC bundles does not increase with increasing MC concentrations but rather seems to decrease while more and more bundles occur. This result is in contrast to the bundling effect caused by PEG where a monotonous increase in the bundle thickness was observed with increasing PEG concentrations [15]. The average thickness of actin/MC bundles can be estimated from phase contrast microscopy pictures depicted in the insets of figure 2 and is on the order of hundreds of nanometers. The obtained thicknesses are comparable to those reported for actin bundles that are induced by depletion forces in the presence of PEG [15] but are considerably larger compared to bundles that are formed by actin bundling proteins such as fascin [16].

As already stated, bundles cannot be observed at MC concentrations below the critical concentration of 0.2% (w/v). In this regime, the contrast is quite low even for confocal images. This is due to the fact that confocal microscopy cannot easily resolve single filaments at high actin concentrations and indicates that only single filaments are present. To overcome the limitations of optical microscopy, we use rheological methods to study the actin/MC gels in more detail.

**Figure 3:**
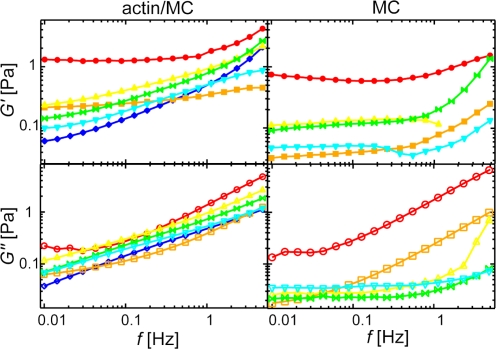
Viscoelastic response of MC and actin/MC solutions. Frequency spectra of storage (upper panel, closed symbols) and loss moduli (lower panel, open symbols) of MC solutions with (left) and without (right) 9.5 µM F-actin. The symbols represent increasing *c*_MC_: 0% (w/v) blue diamonds, 0.01% light blue downright triangles, 0.1% green butterflies, 0.2% yellow upright triangles, 0.5% orange squares and 1% red circles. G′(*f*) and G″(*f*) of actin/MC solutions increase with increasing MC concentration for *c*_MC_≤0.2% (w/v), the critical concentration.

The viscoelastic frequency response of actin gels depends sensitively on the microstructure [17]–[21]. The occurrence of stiff bundles can lead to changes in the deformation mode of the gel [16], attractive interactions between single filaments as induced by entropic depletion forces can serve as virtual cross-links and therefore significantly affect the frequency spectrum of single filaments [15]. The loss modulus, *G*″(*f*), and storage modulus, *G*′(*f*), of pure MC solutions and composite actin/MC gels are shown in figure 3 for distinct MC concentrations, *c*_MC_. The viscous modulus of the MC solutions, *G*″(*f*), is dramatically increased with increasing amounts of MC (fig. 3, right lower panel). However, the viscosity of the solution is not independent from frequency as expected for a purely viscous fluid. Instead, a plateau emerges at low frequencies becoming more pronounced with increasing MC concentrations.

If MC is added to a solution of entangled actin filaments, a qualitative similar behavior is observed for actin/MC gels at low MC concentrations (fig. 3, left lower panel): The *G*″(*f*) spectrum as obtained for pure actin solutions is shifted to higher values in the presence of MC. However, the increase in the loss modulus is not monotonic over the whole concentration range as a significant drop in the loss modulus occurs at *c*_MC_≈0.2% (w/v). This indicates that for a solution of semi-flexible polymers such as actin filaments the presence of MC affects not only the solvent viscosity but might also lead to an interaction with the polymers.

To test this hypothesis the elastic part of the frequency response of actin/MC gels is investigated. The *G*′(*f*) spectra at distinct MC concentrations are shown in figure 3 (left upper panel). In the presence of MC the elasticity is significantly enhanced. Additionally, the elastic spectrum becomes astonishingly flat at high MC concentrations (*c*_MC_>0.2%). Interestingly, the absolute values of *G*′(*f*) of pure MC solutions are on the same order of magnitude as *G*′(*f*) of actin/MC gels, which is consistent with previously published results for pure MC solutions [22]. It is important to note, that the viscoelastic response of MC solutions is highly non-linear as it crucially depends on the applied strain, while the response of actin/MC solutions is linear at low strain. Therefore, the spectra of MC solutions were recorded at the same strain as the corresponding actin/MC gels to allow for direct comparison of the spectra.

In order to quantify the dependence of the storage modulus of actin/MC gels on the MC concentration, the plateau modulus *G*_0_ = *G*′(0.02 Hz) is analyzed (fig. 2). Three different regimes emerge with respect to *c*_MC_: At low MC concentrations (*c*_MC_<0.01% (w/v)) the plateau modulus *G*_0_ is independent from *c*_MC_. At intermediate MC concentrations between 0.01 and 0.2% (w/v) the plateau modulus increases monotonically with *c*_MC_, *G*_0_∼*c*_MC_^0.3^. In the third regime at MC concentrations higher than 0.2% (w/v) the modulus depends on the MC concentration in a non-trivial manner and cannot be described by a simple mathematical function any more.

**Figure 4:**
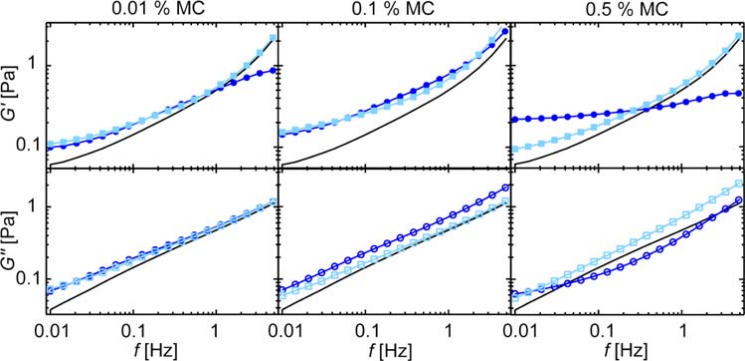
Additive behavior of the viscoelastic response of actin/MC solutions in the intermediate concentration regime. The experimental frequency response of mixed actin/MC solutions (dark blue circles) is compared to the calculated frequency spectrum (light blue squares) assuming additive behavior as described in the text. Frequency spectra of pure actin are shown as a reference (black lines). At intermediate MC concentrations (0.01% and 0.1% (w/v)) the additive behavior holds, while at high MC concentrations significant deviations occur.

In each of the last two regimes the elasticity of the composite material reaches values larger than those of the two constituting components alone. This indicates, that assuming that one component (e.g. the F-actin solution alone) dominates the mechanical response of the composite gel is not sufficient to rationalize the observed viscoelastic response. Another simple approach is to assume, that the polymer systems are mechanically independent from each other resulting in an additive behavior. Indeed, this approximation is valid in the second regime between 0.01 and 0.2% (w/v) MC (fig. 4). This indicates that the structure and mechanical response of entangled actin filaments is not influenced by MC at intermediate concentrations. At higher MC concentrations (>0.2%) the spectra of the two constituents can no longer be summed up to reproduce the mechanical response of the composite material. Here, not only the absolute values of the moduli *G*′(*f*) and *G*″(*f*) are changed but also the shape of the spectra is affected: A flattening of *G*′(*f*) at low frequencies is observed which is unusual for an entangled F-actin solution but is a typical signature of cross-linked or bundled actin gels [16], [19], [23], [24]. This confirms that the structure of the F-actin solution is significantly changed by high MC concentrations.

## Discussion

High concentrations of MC (*c*_MC_>0.2% (w/v)) are able to induce actin bundling as has been shown here by confocal and phase contrast microscopy in agreement with previous studies [11], [12]. This bundle transition correlates well with major changes in the frequency dependent mechanical response of actin/MC solutions. High MC concentrations alter the mechanics of F-actin solutions by an initial decrease in the elastic response. This may result from an effective increase in mesh size caused by bundling of the actin filaments which is supported by confocal images (Fig. 1): At 0.2% MC individual bundles occur while at 0.5% MC a bundle network is observed. The rearrangement of filaments into thick bundles weakens the network elasticity by locally concentrating material and thus creating a network with a huge mesh size. Surprisingly, this trend does not persist throughout the whole MC concentration range and a sudden increase in the plateau modulus is observed at 1% (w/v) MC. Concomitant with this abrupt change in the mechanical behavior a completely different network morphology is observed at this high MC concentration (fig. 1). The observed structural changes agree well with the mechanical properties determined with macrorheology.

The occurrence of actin bundles in the presence of small inert polymers can in principle be attributed to entropic effects known as depletion forces. Entropic actin bundling was reported for actin/PEG 6 k solutions by Tharmann *et al.*, where bundles started to form at 2% (w/w) PEG at 9.5 µM actin [15]. Considering the higher molecular weight of MC (88 kDa) the corresponding bundling transition should occur at roughly 5% (w/v) MC. However, the critical bundling concentration for MC is an order of magnitude lower compared to this calculated value. This indicates that additional contributions to the interaction potential between actin filaments might occur in the MC system. In particular, the presence of the MC network might impede the formation of a fully equilibrated actin network structure. As a consequence, kinetic trapping effects might play a role [25] and might explain the occurrence of thinner bundles at high MC concentration.

More subtle changes in the viscoelastic behavior of actin/MC solutions are induced by intermediate MC concentrations (between 0.01 and 0.2% (w/v)). Here, no bundles can be detected by phase contrast and confocal microscopy. In this regime, the enhancement of the network elasticity can roughly be approximated by a power law, *G*_0_∼*c*_MC_^0.3^. A similar regime showing a weak increase in the network elasticity has been determined for actin/PEG solutions below the bundling transition. There, *G*_0_∼*c*_PEG_^0.2^ was observed and attributed to increasing attractive forces between single filaments upon addition of depletion agent [15]. However, a similar scaling law does not directly imply that the same molecular mechanisms are responsible for the enhancement of the elasticity. Recall, that MC solutions themselves show a viscoelastic behavior which is not observable for PEG solutions at these low concentrations. In marked contrast to purely viscous fluids the loss modulus of MC solutions does not follow *G*″(*f*) = *η*/(2*πf*) (fig. 3) and a significant elastic response is observed. Surprisingly, the absolute values of *G*″(*f*) as well as *G*′(*f*) are on the same order of magnitude as the viscoelastic response of F-actin solutions alone. This suggests that MC concentrations as low as 0.01% (w/v) might already be beyond the overlap concentration creating an entangled viscoelastic material. In fact, the critical concentration of a slightly smaller MC was found to be 0.03% (w/v) [26] which corresponds well to our findings considering the small differences in the molecular weight.

Due to the significant viscoelastic properties of pure MC solutions themselves, in this intermediate MC concentration regime both components, the F-actin and the MC solution, contribute to the mechanical response of the composite material – at least in the elasticity dominated frequency regime investigated in this study. The structural organization of the actin solution seems not to be altered by low concentrations of MC. Thus, the elastic regime between 10 mHz and 5 Hz will be dominated by the entanglements of both polymer solutions and no cooperative influence on the elasticity (or the viscous dissipation) in this low frequency regime would be expected. This gives rise to the simple additive behavior that is observed for both viscoelastic moduli. Matters might be different at higher frequencies (typically around ∼1 kHz and above) where Rouse-like fluctuations of single filaments in a viscous medium determine the viscoelastic response of an entangled actin solution [27], [28] or cross-linked actin networks [21]. At such high frequencies the presence of MC might very well have additional effects on the frequency response of the actin solution which could defy a simple addition of distinct frequency spectra in this regime.

However, in the experimentally more relevant frequency regime <5 Hz the observed mechanical additivity provides a simple method to derive the mechanical properties of a polymer solution from a polymer/MC mixture. Using a simple calibration measurement of the pure MC solution, the viscoelastic response of MC can be subtracted from the response of the polymer/MC solution. In contrast, due to structural changes at MC concentrations *c*_MC_>0.2% (w/v) this strategy is not applicable in the high concentration regime.

In conclusion, methylcellulose is not suitable to simply increase the viscosity of polymer solutions as MC can significantly alter the structure and mechanics of entangled F-actin solutions at concentrations higher than 0.2% (w/v) MC. Theses alterations are observed over the whole elasticity dominated frequency regime studied here which also represents the time frame of typical biological and biochemical experiments. Therefore, MC should very cautiously be used to determine biochemical processes that take place on comparable time scales. In the intermediate concentration regime, the contribution of MC can be subtracted from the frequency spectra of the storage and loss modulus. This is no longer possible above the critical concentration, as the structural changes induced by MC defy a simple analysis of the mechanical properties of the bundled network. However, below the critical concentration the solvent viscosity cannot be increased sufficiently which drastically limits the utility of MC.

## Materials and Methods

### Protein preparation

Actin is prepared from rabbit skeletal muscle [29] and stored in lyophilized form at -20°C. For measurements the lyophilized actin is dissolved in deionized water and dialyzed against G-buffer (2 mM Tris (pH 8), 0.2 mM ATP, 0.2 mM CaCl_2_, 0.2 mM DTT and 0.005 % NaN_3_) at 4°C. The G-actin solutions are stored at 4°C and used within seven days of preparation. The average length of the actin filaments is controlled by adding gelsolin which is isolated from bovine plasma serum [30], [31].

### Sample preparation

Samples are prepared by gently mixing deionized water with gelsolin and G-actin and buffered to 2 mM Tris/HCl (pH 7.5), 2 mM MgCl_2_, 0.2 mM CaCl_2_, 0.2 mM DTT, 100 mM KCl and 0.5 mM ATP. Methylcellulose (Sigma M0512, approx. 88 kDa, viscosity 4000 cP if dissolved 2% (w/v) in H_2_O) is dissolved in deionized water to 2% (w/v) or less and added to the sample before actin polymerization was induced. An actin concentration of 9.5 µM, an average filament length of 21 µm and a measuring temperature of 21°C is used. For experiments with pure MC solutions deionized water is used instead of G-buffer.

### Rheology

Approximately 460 µL sample volume are loaded into a commercial stress controlled rheometer (Physica MCR301, Anton Paar, Graz, Austria) with a 50 mm plate-plate geometry and 160 µm plate separation. During polymerization the storage modulus G′(0.5 Hz) is monitored to ensure completed polymerization before measurements. Oscillatory measurements are performed with constant strain ranging from 1.5% to 10%, depending on the sample rigidity.

### Phase contrast microscopy

Samples prepared as described above were filled into microscope chambers prior to polymerization. 1 h after the polymerization was initiated, the samples are investigated by phase contrast microscopy using a 63× oil immersion objective (1.4 numerical aperture).

### Confocal microscopy

The samples were prepared as described for phase contrast microscopy. However, actin filaments were stained by adding 0.95 µM phalloidin-tetramethylrhodamine (Sigma) prior to polymerization. Images were taken 1 h after polymerization was induced using a Leica TCS SP5 confocal microscope. Images were processed with Leica Microsystems LAS AF software and show maximal intensity projections of either time series (for 0%–0.1% MC samples) or z-stacks (for 0.2%–1% MC samples).
